# Assessing the impact of dental restorative materials on fibroblast cells: an immunohistochemical and ELISA analysis

**DOI:** 10.1038/s41598-024-54331-2

**Published:** 2024-02-27

**Authors:** Mustafa Duzyol, Pinar Bayram, Esra Duzyol, Selina Aksak Karamese

**Affiliations:** 1 Department of Restorative Dentistry, Faculty of Dentistry, Istanbul Medeniyet University, Istanbul, Turkey; 2 Department of Histology and Embryology, Faculty of Medicine, Kafkas University, Kars, Turkey; 3 Department of Pediatric Dentistry, Faculty of Dentistry, Istanbul Medeniyet University, 34200 Istanbul, Turkey

**Keywords:** Fibroblast, Dental filling materials, Inflammation, Oxidative stress, Apoptosis, Biochemistry, Immunochemistry

## Abstract

In this study, our aim was to investigate the effects of restorative materials such as composite, compomer and high viscosity glass ionomer, which are frequently used in dentistry, on L929 fibroblast cells by evaluating the oxidative stress parameters, pro- and anti-inflammatory cytokines, and apoptosis markers. L929 fibroblast cells were cultured, and dental filling materials were applied in two doses (50 and 100 µl). Immunohistochemical staining was performed for experimental groups with Anti-Bax and Anti-Caspase 9 antibodies. Then, ELISA technique was used to detect the level of TNF-alpha, TGF-beta, IL-1-beta, IL-6, IL-10, LPO and CAT. In the light of the data, the examined dental filling materials were effective on increasing the TGF-beta, IL-10, LPO and CAT levels, and decreasing the TNF-alpha, IL-1-beta, and IL-6 levels. The histological micrographs were also support the issues. When the levels of H-score in Caspase 9 labeled micrographs were evaluated, the mean of the control group was lower than the mean of the experimental groups. Biocompatibility varies according to the content of the material, the amount of residual monomer, and its solubility. Although all the experimental groups have cytotoxic effects, the least effect is seen in the Omnichroma group.

## Introduction

According to the developing technology in dentistry, restorative materials with different properties are produced. Restorative materials must be biocompatible with oral tissues^1–5^. While evaluating restorative materials, the biologically harmful effects of these materials should be determined. Composites and compomers, one of the restorative materials used in dentistry, include different methacrylate groups such as urethane dimethacrylate (UDMA), bisphenol A-glycidyl methacrylate (Bis-GMA), hydroxyethyl methacrylate (HEMA), and triethylene glycol dimethacrylate (TEGDMA)^6–10^. There is a strong link between the clinical success of dental materials and their biological compatibility^11^. Studies have reported that restorative materials have adverse effects on periodontal tissues, depending on the type of restorative material, the presence of protrusions and surface roughness, and the location of the restoration on the tooth surface (for example, sub or supragingival)^12–14^.

The cytotoxicity of dental composites is tightly linked to the release of residual monomers due to degradation processes or incomplete polymerization of materials^15^. Due to the low amounts of compounds released from resin-based materials into aqueous solutions, it is clear that processes except acute cytotoxicity are of paramount importance for the interpretation of cell responses. The ability to alter essential cellular functions beyond acute cytotoxic concentrations has led, for example, to the discovery of the induction of heat shock proteins to compensate for significant protein damage, the discovery of modifications of immune system cells or cellular responses to damage by underlying genetic effects such as the induction of gene mutations or chromosomal aberrations. Residual monomers are blamed for the cytotoxicity of restorative materials^16^. These residual monomers dissolve with agents such as oral liquids or fluids in the dentinal tubules, affecting the soft tissues of the oral cavity and the dentin-pulp complex^6–10^. Recently, in vitro, and in vivo studies have turned to revealing the cytotoxic, genotoxic, cellular reactive oxygen production-enhancing and general health-impacting properties of residual monomers.

Studies have shown that compounds released from restorative materials can increase bacterial growth and glutathione consumption, which is important in pulp and gingival cell apoptosis and production of reactive oxygen products^16,17^. As a result, changes occur in the levels of some cytokines such as interleukin-1-β (IL-1-β), interleukin-6 (IL-6) and tumor necrosis factor alpha (TNF-α). Cytokines play an important role in inflammatory response, tissue destruction in periodontal diseases and regulation of adaptive immune response^18,19^.

In this study, our aim was to investigate the effects of restorative materials such as composite, compomer and high viscosity glass ionomer, which are frequently used in dentistry, on L929 fibroblast cells by evaluating the oxidative stress parameters, pro- and anti-inflammatory cytokines, and apoptosis markers.

## Materials and methods

### Experimental groups

The experimental groups and the detailed information about dental filling materials and applicated doses are in Table 1.

**Table 1:** The experimental groups of our study.

Group codes	Doses	Materials	Composition	Manufacturer
Cnt	None	Control Group		
A100	100 µl	Equıa Forte	Fluoroaluminosilicate glass, polybasic carboxylic acid, polyacrylic acid, water, iron oxide	GC Corporation, Tokyo, Japan
A50	50 µl			
B100	100 µl	Dyract AP	UDMA (Urethane dimethacrylate), iron oxide pigments. TCB resin (tetracarboxylic acid-hydroxyethylmethacrylate-ester), butyl hydroxy toluene alkanoyl-poly-methacrylate, strontium fluoride strontium-fluoro-silicate glass, photo initiators	Dentsply De Trey, Konstanz, Germany
B50	50 µl			
C100	100 µl	Estelite P Quick	TEGDMA, 2-Propenoic acid, 2-methyl-, (1-methylethylidene, bis[4,1-phenyleneoxy(2-hydroxy-3,1-propanediyl)] ester, titanium dioxide, 2,6-di-tert-butyl-p-cresol; p-methoxyphenol	Tokuyama Dental Corporation, Tokyo, Japan
C50	50 µl			
D100	100 µl	Omnichroma	UDMA/TEGDMA monomers, spherical SiO_2_-ZrO_2_	Tokuyama Dental Corporation, Tokyo, Japan
D50	50 µl			
E100	100 µl	Filtek Z250	Bis-GMA (bisphenol glycidylmethacrylate), non-agglomerated silica nanoparticles UDMA (urethane dimethacrylate), bis-EMA (ethoxylated bisphenol dimethacrylate), TEGDMA (triethlene glycol dimethacrylate	3M ESPE, St Paul, MN, USA
E50	50 µl			
F100	100 µl	SureFil SDR flow	Modified UDMA, bis-EMA, TEGDMA	Dentsply DeTrey, Konstanz, Germany
F50	50 µl			

### Extract preparation

This study was organized according to the standards of the International Organization for Standardization. (ISO) no. 10993-5:20097 and 10993-12:2021.

After Equia Forte (GC Corporation, Tokyo, Japan) was mixed in an amalgamator, it was placed in a 0.5 mm thick, 6 × 10 cm Teflon mold. The restorative material mixing time was 10 s and the setting time was 2 min 30 s after placing into the cavity. Afterwards, eqiua coat was applied and cured with light for 20 s using the Valo LED (8 mm optical diameter) curing light (Ultradent, South Jordan, USA) standart mode (1200 mW/cm^2^) keeping it 7.5 mm away from the material surface. In the compomer and composite groups, they were placed in a 0.5 mm thick, 6 × 10 mm Teflon mold and polymerized in accordance with the manufacturer's recommendations for 20 s using the Valo LED curing light (Ultradent, South Jordan, USA) keeping it 7.5 mm away from the material surface. For cytotoxicity testing, samples were placed in a 50 ml extraction flask containing 60 cm^2^ of test substance covered with 20 ml of minimal basic medium. After aging for 24 h at 37°, 50–100 µl of solution samples were taken and applied.

### Cell culture protocol

L929 fibroblast cells was purchased from the American Type Culture Collection (ATCC, USA) and were cultured in Dulbecco's modified eagle's medium (DMEM) with 10% fetal bovine serum (FBS)and 2% penicillin/streptomycin solutions in a T75 cell culture flask. After sufficient confluence was achieved, cells were detached from the flask Trypsin–EDTA solution (Gibco, Pittsburgh, USA) and counted with trypan blue (Sigma-Aldrich, USA) under an invert microscope (Zeiss Axio). Cells were seeded on 24-well plates for immunohistochemical analysis and 96-well plates for cell viability assay and ELISA assay. The cells were incubated for 24 h at 37 °C in an atmosphere of 5% CO_2_.

### Cell viability analysis

For the cell viability and proliferation analysis, Cell Viability Detection Kit-8 (CVDK-8; EcoTech Biotechnology) assay was performed. In the tissue culture medium, CVDK-8 is reduced by dehydrogenases in cells to give an orange color of formazone. For this assay, 10^4^ cells per well in a 96 well plate in 100 µl DMEM was seeded and incubated for 24 h. Then, drugs were added and incubated for 24 h. 10 µl CVDK-8 was added per well and incubated at 37 °C in a humidified 5% CO_2_ incubator for 3 h. The absorbance was measured using a spectrophotometric plate reader (Multiskan GO, Thermo Scientific) at a wavelength of 450 nm. All experiments were done in duplicate in at least three cultures.

### Immunohistochemical analysis

A cover-glass was placed on the bottom of the 24 well plates and 4 × 10^4^ cells seeded in each plate. When sufficient confluence was provided, drugs were added in indicated doses. After 48 h, the medium was removed and washed with PBS and cells fixed with 10% buffered formalin. To remove the formalin, cells were washed with PBS and incubated with blocking solution (Thermo Scientific, TA-060-PBQ) for 5 min. Then blocking solution was removed and cells incubated overnight with primary antibodies anti-Bax (Bioscience, FNab00081) and anti-Caspase 9 (Santa Cruz, SC-70506) at + 4 °C. And then primary antibodies were removed, washed with PBS, and incubated with primary antibody enhancer (Thermo Scientific, TA-060-PB) for 10 min. After washed with PBS, cells incubated with HRP polymer (Thermo Scientific, TA-060-PH) for 10 min and washed with PBS. 3,3-diaminobenzidine (DAB) solution (Thermo Scientific, TA-060-HDX) was applied on the cells for 2 min, the counterstaining was done with Harris Hematoxylin. The cells were dehydrated, and the cover-glass was taken on the slide and mounted with Entellan.

For immunohistochemical analysis, the slides photographed under 20× magnifications using a light microscope (Olympus BX43 with DP21 camera system). Micrographs were analyzed in ImageJ software (ImageJ1, 51j8, National Institutes of Health, USA) to determine staining intensities (i) which were determined as negative: 0, low positive: 1, positive: 2, high positive: 3. Pixel ratios (Pi) were determined according to the staining intensities between 0 and 100%. H-SCORE rates were calculated using the obtained values formula. H-SCORE = Σ Pi (i + 1)^20^.

### ELISA technique

To detect the levels of oxidative stress parameters and the pro- and anti-inflammatory response of L929 cells after exposure to dental filling materials, we performed ELISA technique. We used Human Tumor Necrosis Factor alpha (TNF-alpha, BT Lab, Bioassay Technology Lab, Zhejiang, China, Cat No: E0082Hu), Human Interleukin-6 (IL-6, BT Lab, Bioassay Technology Lab, Zhejiang, China, Cat No: E0090Hu), Human Interleukin-1-beta (IL-1-beta, BT Lab, Bioassay Technology Lab, Zhejiang, China, Cat No: E0143Hu), Human Catalase (CAT, BT Lab, Bioassay Technology Lab, Zhejiang, China, Cat No: E3053Hu), and Human Super Oxidase Dismutase (SOD, BT Lab, Bioassay Technology Lab, Zhejiang, China, Cat No: E0918Hu) ELISA Kits according to the manufacturer instructions.

### Statistical analysis

The results were expressed as mean ± SEM. The statistical difference among the groups was evaluated by One-Way ANOVA, the statistical difference between for more than 2 independent numerical data was evaluated by Tukey test. All data were analyzed using GraphPad Prism, version 5.0 for Windows (Graph Pad Software, San Diego, California, USA). The level of p < 0.05 was considered statistically significant.

## Results

### Immunohistochemical findings

**Figure 1:**
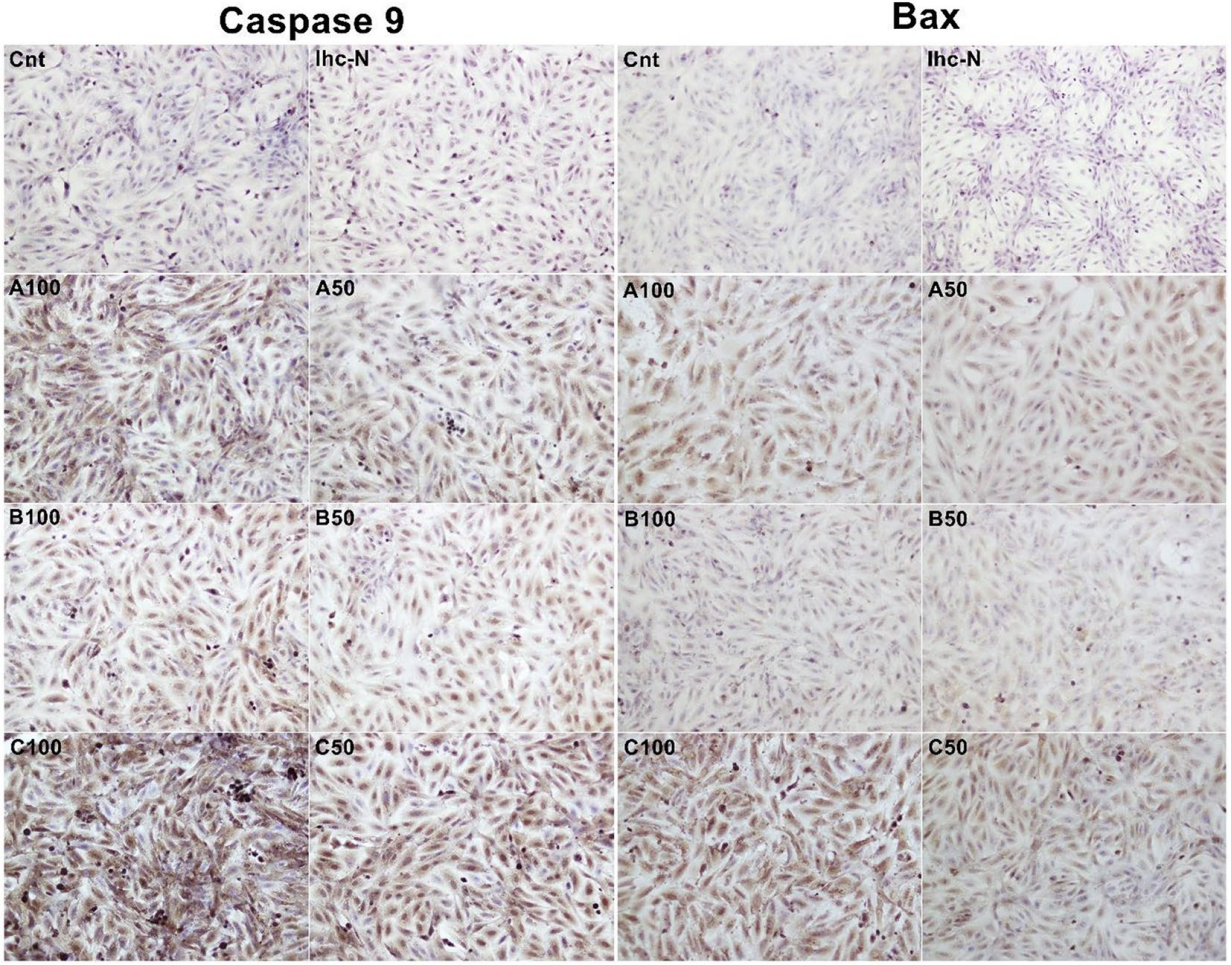
Bax and Caspase-9 expressions in L929 cell line, brown staining indicates positive immune-reactivity. First and second columns are showing Caspase-9 staining at ×20 magnification; third and fourth columns are showing BAX staining at ×20 magnification. Cnt, control; Ihc-N, negative control of IHC; A100, incubation with 100 µl Equia Forte; A50, incubation with 100 µl Equia Forte; B100, incubation with 100 µl Dyract AP; B50, incubation with 100 µl Dyract AP; C100, incubation with 100 µl Estelite P Quick; C50, incubation with 100 µl Estelite P Quick.

**Figure 2:**
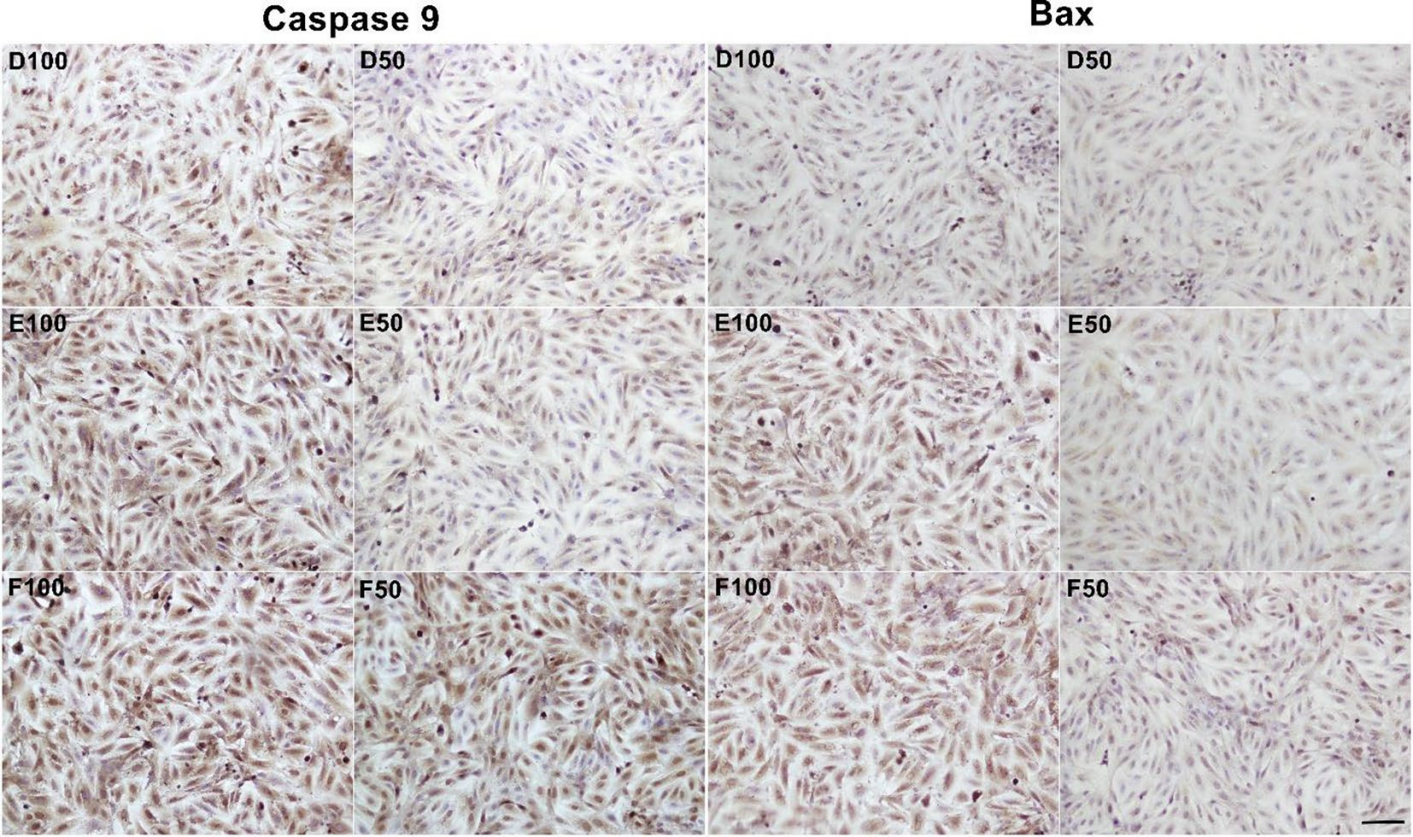
Bax and Caspse-9 expressions in L929 cell line, brown staining indicates positive immune-reactivity. First and second columns are showing Caspase-9 staining at ×20 magnification; third and fourth columns are showing BAX staining at ×20 magnification. Cnt, control; Ihc-N, negative control of IHC; D100, incubation with 100 µl Omnichroma, D50, incubation with 50 µl Omnichroma; E100 incubation with 100 µl Filtek Z250, E50 incubation with 50 µl Filtek Z250, F100 incubation with 100 µl SureFil SDR flow, F50 incubation with 50 µl SureFil SDR flow.

Different doses of dental filling materials were applied to L929 cells. Immunohistochemical staining was performed by Bax and Caspase 9 primary antibodies to determine apoptosis levels (Figs. 1 and 2).

**Figure 3:**
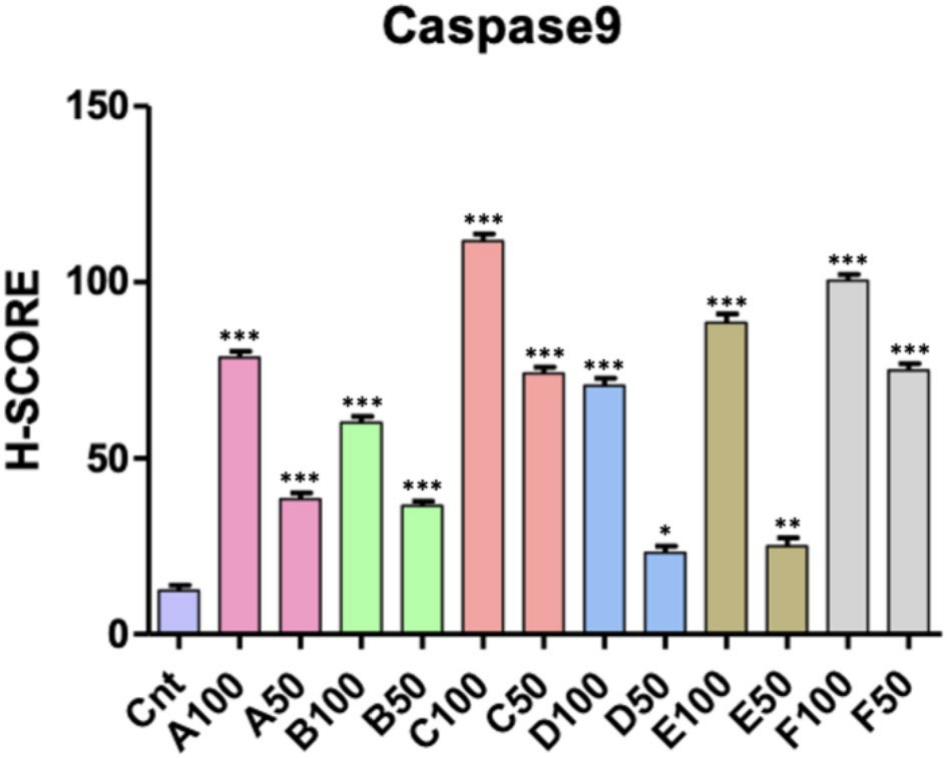
The mean H-score levels of immunohistochemical labeling of Caspase 9. *Control versus all experimental groups; *p < 0.05, **p < 0.01, ***p < 0.001.

In our study, when the levels of H-score in Caspase 9 labeled micrographs were evaluated, a significant increase was observed among the groups (p < 0.0001). When the control group and experimental groups were compared in terms of Caspase 9 expressions, a statistically significant increase was observed in all groups. It also was determined a statistical differences between the H-score levels of same compounds high dose (100) and low dose (50) (p < 0.001). When the apoptosis levels between the experimental groups were compared, it was determined that the H-score values of C100 and D50 were the highest and the lowest respectively (Fig. 3).

**Figure 4:**
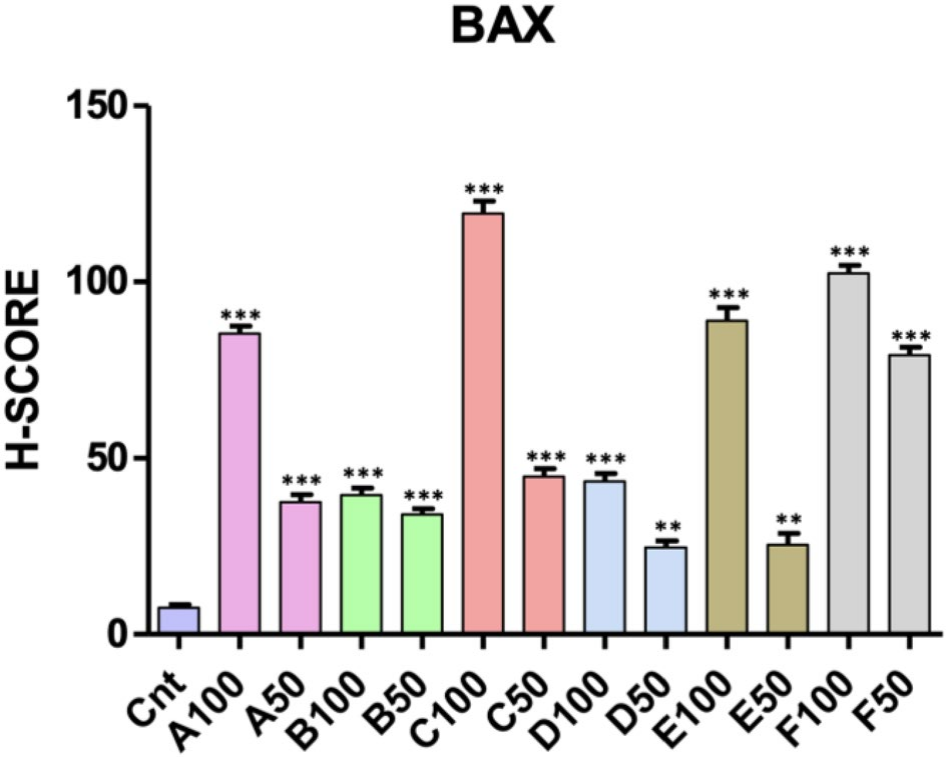
The mean H-score levels of immunohistochemical labeling of Bax. * Control (Cnt) versus all experimental groups; **p < 0.01, ***p < 0.001.

When the levels of H-score in Bax labeled micrographs were evaluated, a significant increase was observed among the groups (p < 0.0001) moreover these levels were similar to Caspase 9-labeled micrographs. It also was determined a statistical differences between the H-score levels of same compounds high dose (100) and low dose (50) (p < 0.001) except B gruop. (Fig. 4). Similarly to the Caspase 9 results, it was determined that the H-score values of C100 and D50 were the highest and the lowest respectively.

### ELISA findings

**Figure 5:**
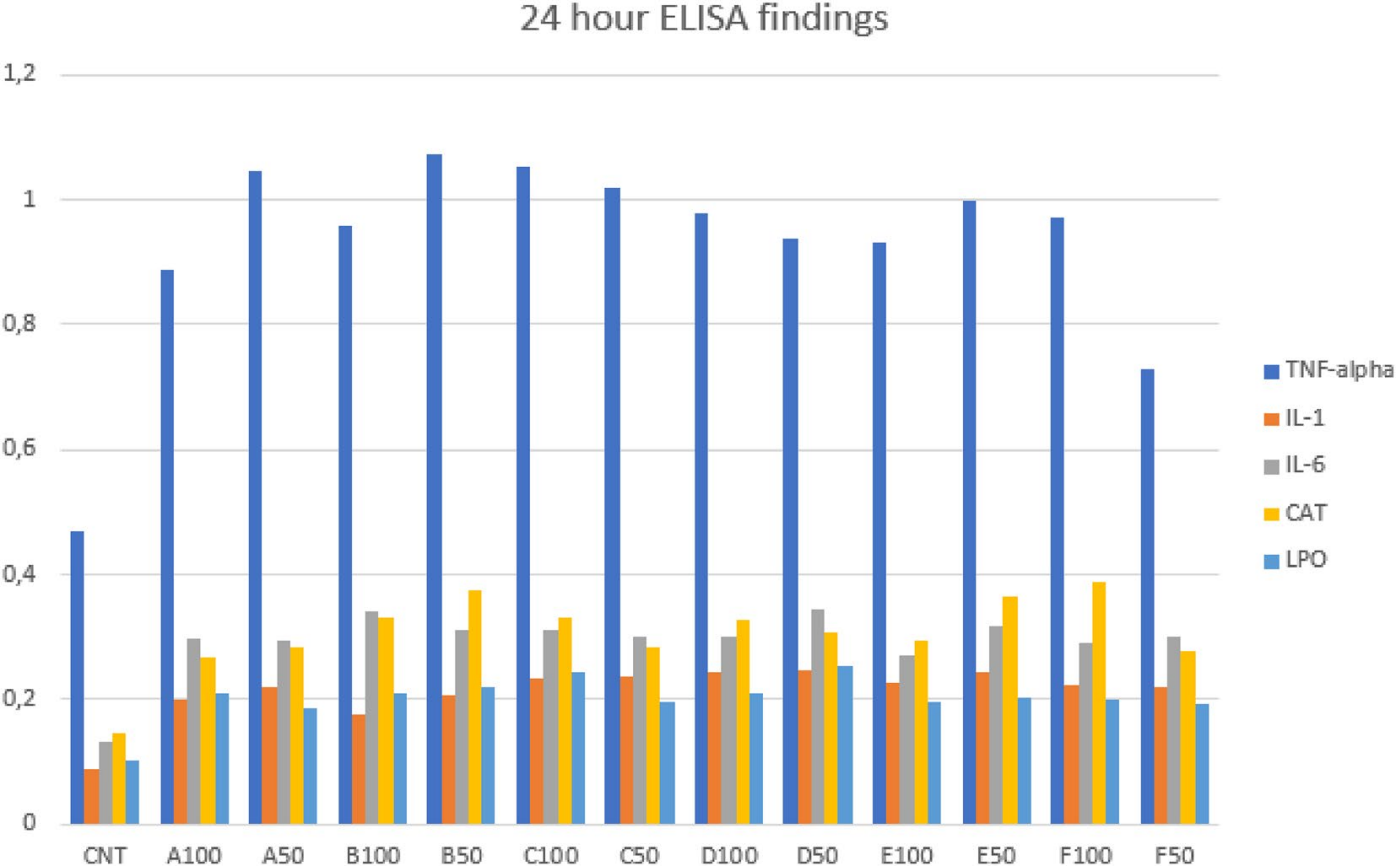
The 24-h cytokine levels of experimental groups.

After the dental filling materials were applied to L929 cells, we incubated them both 24 and 48 h and observed the changes in some cytokine and oxidative stress parameters levels. As seen in Fig. 5, all cytokine and oxidative stress parameters were higher, when compared to control group level. The dental filling materials that trigger the most cytokine and oxidative stress parameters release were Dyract AP, Estelite P Quick, Equia Forte, Filtek Z250, Omnichroma, and SureFil SDR flow respectively, according to the 24-h results.

**Figure 6:**
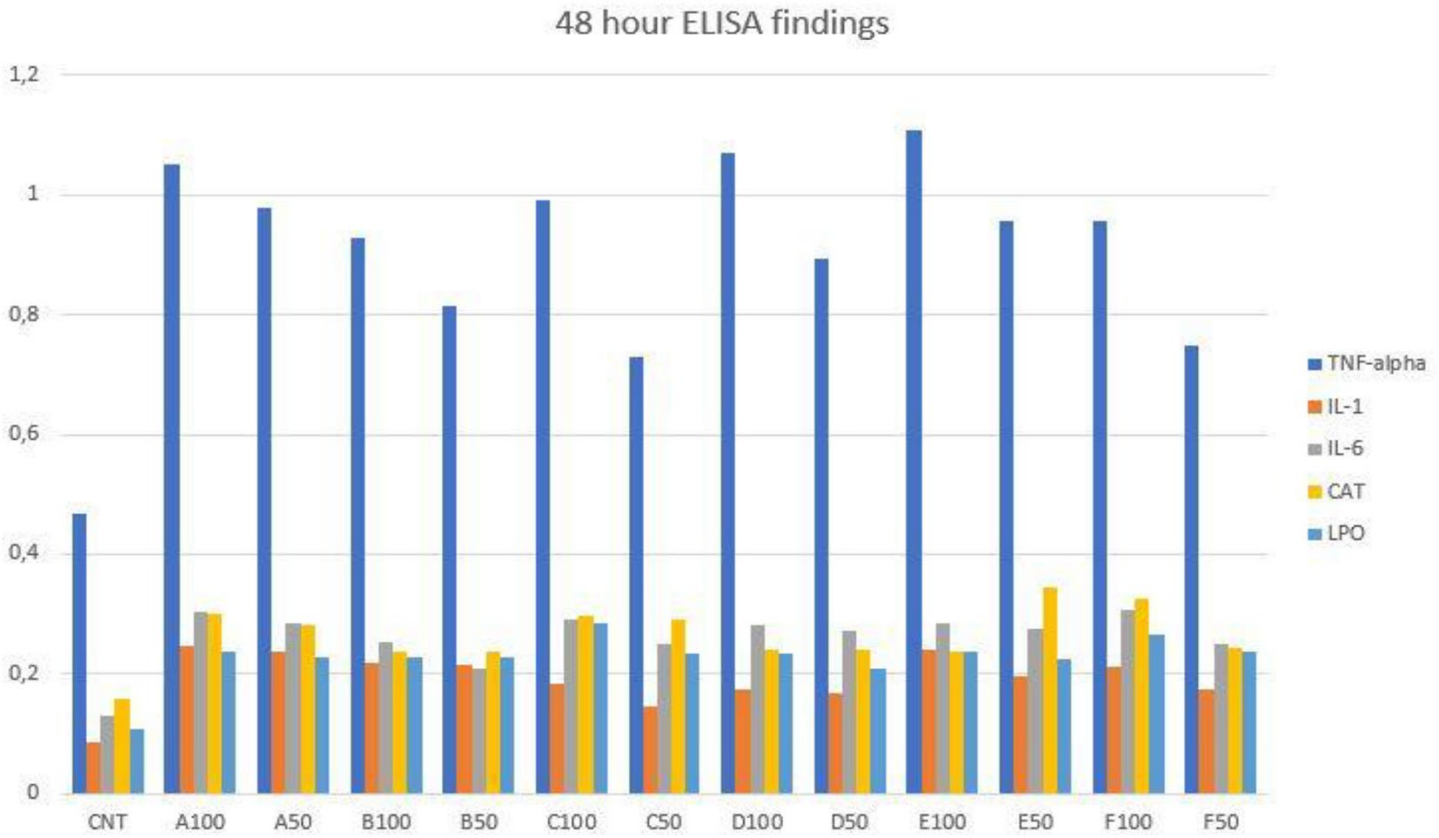
The 48-h cytokine levels of experimental groups.

Additionally, the 48-h findings were almost the same with 24-h findings. all cytokine and oxidative stress parameters were higher, when compared to control group level (Fig. 6). However, the filling material order was different in terms of triggering the release of cytokine and oxidative stress parameters. The order was Filtek Z250, Omnichroma, Estelite P Quick, Equia Forte, Dyract AP and SureFil SDR flow respectively.

## Discussion

In restorative dentistry, the increasing aesthetic and cosmetic demands of the patients and the preference of non-invasive procedures have made the use of composite materials widespread. These resin-containing materials contain various monomers in different proportions. These monomers are generally monomers such as propane (Bis-GMA), urethane dimethacrylate (UDMA), 2-hydroxyethylmethacrylate (HEMA) and triethylene glycol dimethacrylate (TEGDMA)^3^. Monomers are known to be effective on the cytotoxic effects of materials. Dental materials must be evaluated in terms of biological risks before their clinical use.

Different techniques and methods are used by researchers while investigating the cytotoxic effects of materials. In-vitro tests, compared to other biocompatibility tests; In vitro tests were also used in this study because they have important advantages such as being able to be concluded in a short time, being less costly than animal experiments or clinical use tests, being controllable and standardizable, and being well adapted to wide-ranging screening^15^.

The presence of high levels of reactive radicals such as hydroxyl, hydrogen peroxide and superoxide, which occur because of normal aerobic metabolism reactions in cells, cause an increase in oxidative stress in cells and tissues and cause cellular damage^21^. Oxidative stress is the condition that occurs when metabolic prooxidant production exceeds antioxidant capacity^22^. Measurement methods based on oxidative stress appear to play an important role in determining the biocompatibility of a material. Therefore, oxidative stress measurement was performed in our study.

Experimental details such as sample preparation modes, curing conditions for mixed materials, aging of samples, and preparation of extracts in various solvents greatly influence the cytotoxic effects of dental materials in mammalian cell cultures^23^. In a study^15^, various aging periods after the curing of dental composite materials and variations of exposure periods of mammalian cell cultures were analyzed in terms of their effects on the cytotoxicity of material extracts and showed that cytotoxicity was independent of aging periods. In our study, aging was applied for 24 h at 37°.

This study was aimed to compare the effect of composites with different monomers and glass ionomer-based restorative materials on L929 fibroblasts in vitro. Although there are limited resources on this subject in the literature, it has not been fully revealed how the contents of the material create cytotoxic effects. Similar to the results found by Beltrami et al.^24^, Omnichroma group gave the least cytotoxic effect at low doses in our study. However, Dyract AP caused the least cell apoptosis at high doses. Also, more apoptosis was observed in high doses (100) groups than in low (50) groups. Among the experimental groups, the highest apoptosis value was Estelite P Quick (100), and the lowest value was Omnichroma (50). In addition to cell viability rates were highest in the control group, respectively, D50, E50, B50, A50, B100, D100, C50, F50, A100, E100, F100 and C100. Kamalak et al.^25^ reported a correlation between filler content and cytotoxicity. According to the current literature, cytotoxicity increases as the amount of filler increases. In parallel with this result in our study, Estelite, which has the highest filling rate with 82%, also has the highest cytotoxicity Studies in the literature show that methacrylate-containing restorative materials are more cytotoxic. In our study, it was observed that Dyract AP, which has many different methacrylate group monomers, caused more cellular response and oxidative stress at the end of 24 h. After 48 h, Filtek Z250 was found to cause the most cellular response and oxidative stress.

The results of our study should be supported by contact in vitro tests and in vivo tests in future studies. All the restorative materials with different contents and chemical structures used in our study have been shown to have cytotoxic effects on fibroblasts. However, their potential to cause chronic periodontal problems should be investigated by performing long-term tests.

## Conclusions

Our study shows that biocompatibility cannot be explained by looking at a single reason. Biocompatibility varies according to the content of the material, the amount of residual monomer, and its solubility. Although all the experimental groups have cytotoxic effects, the least effect is seen in the Omnichroma group. Further detailed studies will be performed to try to solve the molecular mechanisms of this cellular responses by examining some crucial parameters including Caspase-3.

## Data Availability

The datasets used and/or analysed during the current study available from the corresponding author on reasonable request.
